# DTL Is a Prognostic Biomarker and Promotes Bladder Cancer Progression through Regulating the AKT/mTOR axis

**DOI:** 10.1155/2022/3369858

**Published:** 2022-01-21

**Authors:** Yongwen Luo, Zhiwen He, Wei Liu, Fenfang Zhou, Tao Liu, Gang Wang

**Affiliations:** ^1^Department of Urology, Zhongnan Hospital of Wuhan University, Wuhan, China; ^2^Department of Biological Repositories, Zhongnan Hospital of Wuhan University, Wuhan, China; ^3^Human Genetic Resource Preservation Center of Hubei Province, Wuhan, China; ^4^Human Genetic Resource Preservation Center of Wuhan University, Wuhan, China; ^5^Laboratory of Precision Medicine, Zhongnan Hospital of Wuhan University, Wuhan, China; ^6^Wuhan Research Center for Infectious Diseases and Cancer, Chinese Academy of Medical Sciences, Wuhan, China

## Abstract

**Background:**

Denticleless E3 ubiquitin protein ligase homolog (DTL) has been reported to be an important regulator for tumorigenesis and progression. Nonetheless, the biological functions and molecular mechanisms of DTL in BCa remain elusive.

**Methods:**

We implemented integrative bioinformatics analysis to explore the diagnostic and prognostic values of DTL based on The Cancer Genome Atlas (TCGA), ArrayExpress, and Gene Expression Omnibus (GEO) databases. Then, we utilized qRT-PCR and immunohistochemistry to verify the clinical significance of DTL expression according to clinical specimens and tissue microarray (TMA). Moreover, the biological functions and underlying mechanisms of DTL in BCa were investigated through in vitro and in vivo experiments.

**Results:**

Integrative bioinformatics analysis revealed that DTL was a key gene associated with BCa progression, and increased DTL expression was correlated with malignant biological behavior and poor prognosis. Experiments on clinical specimens and tissue microarray (TMA) further confirmed our findings. Bioinformatics analysis demonstrated that DTL could be associated with cell cycle- and DNA replication-associated pathways in BCa. The suppression of DTL inhibited BCa cell proliferation, migration, and invasion in vivo and in vitro. Mechanistically, DTL may promote BCa progression through the AKT/mTOR pathway.

**Conclusions:**

Increased DTL expression was correlated with malignant biological behavior and poor prognosis of BCa patients, and it may promote BCa progression through the AKT/mTOR pathway. Our research provided a potential predictor and therapeutic target for BCa.

## 1. Introduction

Bladder cancer (BCa) is a malignant tumor that originates from the bladder mucosa, and the global incidence is increasing annually. According to global cancer statistics in 2020 [1], there are approximately 573,278 new BCa cases worldwide, accounting for about 3% of all new tumor cases, and 212,536 BCa deaths occurred in 2020, accounting for 2.1% of all cancer deaths. To date, the treatment of BCa was limited to surgery and to immunotherapy or chemotherapy due to the lack of precise molecular targets for BCa [2, 3], whereas the majority of BCa patients still have poor prognosis after systemic therapy [4, 5]. Therefore, it is particularly important to explore the occurrence and development mechanisms of BCa and discover novel candidate biomarkers to improve the early diagnosis and treatment outcomes.

Nowadays, with the development of high-throughput gene chip and next-generation sequencing technology, a number of gene expression profiles have been used for biological study of various cancers [6, 7], and they are stored in some public genomics data repositories open to public. Researchers can integrate and analyze these datasets and discover potential key biomarkers for cancer diagnosis and prognosis. For example, our previous study identified nine key genes associated with progression of renal cell carcinoma, including PTTG1, RRM2, TOP2A, UHRF1, CEP55, BIRC5, UBE2C, FOXM1, and CDC20 [8]. And some of them have been experimentally verified by many researchers [9–11].

Currently, weighted gene coexpression network analysis (WGCNA), a method of the coexpression module correlation analysis across microarray samples, has been widely used to identify the hub genes associated with clinical characteristics by constructing scale-free gene coexpression networks [12–14]. During the network construction, highly coexpressed genes are connected in the network and divided into modules. Then, the most central and connected genes in different modules with different functionally related genes are treated as hub genes. It is reported that WGCNA was proven to be a promising and reliable tool for validation of key genes associated with disease pathogenesis.

In this study, we performed WGCNA to explore key oncogenic factors associated with BCa oncogenesis and progression using publicly available gene expression data, we found that DTL is a critically potential diagnostic and prognostic oncogene, our tissue microarray data validated that DTL was an independent prognostic factor for unfavorable overall survival of BCa patients. By in vitro and in vivo assays, we demonstrated that the suppression of DTL in BCa cells significantly inhibits cell proliferation, invasion, and migration capacity; mechanistic studies revealed that DTL may modulate cell proliferation and induce epithelial-mesenchymal transition (EMT) through activation of the AKT/mTOR signaling pathway. Our results indicate that DTL may be a potential target for preventing and treating BCa.

## 2. Materials and Methods

### 2.1. Study Cohort and Data Preprocessing

BCa mRNA expression profile data and corresponding clinicopathological information were obtained from four publicly available datasets, TCGA-BLCA, E-MTAB-4321, GSE13507, and GSE32894. The microarray datasets of GSE13507 and GSE32894 were downloaded from the GEO database (https://www.ncbi.nlm.nih.gov/geo/) on the Illumina platform. The dataset E-MTAB-4321 was obtained from the ArrayExpress database (https://www.ebi.ac.uk/arrayexpress) on the Illumina platform. The RNA-seq data of the TCGA-BLCA cohort were downloaded using the R package “TCGAbiolinks.” The “EdgSeq” R package was used in the normalization of mRNA-seq data, while the “limma” R package was employed to process the gene microarray data.

### 2.2. Weighted Gene Coexpression Network Construction

Scale-free gene coexpression networks were constructed by using the “WGCNA” R package [12]. Briefly, gene expression profiles were tested to check if they were good samples and good genes. Pearson's correlation matrices were constructed for all pair-wise genes. A weighted adjacency matrix was then established utilizing a power function amn = |cmn|^*β*^ (cmn is Pearson's correlation between gene *m* and gene *n*; amn is the adjacency between gene *m* and gene *n*). *β* represents a soft thresholding parameter that could emphasize the strong correlations between genes and penalize weak correlations. Next, the adjacency was transformed into a topological overlap matrix (TOM). To classify genes with similar expression profiles into gene modules, average linkage hierarchical clustering was performed in accordance with TOM-based dissimilarity measurement, with a minimum size of 50 for the gene dendrogram. To confirm the key modules and genes, we set the module membership (MM) and gene significance (GS) to be the measure used to identify the correlation between genes and clinical phenotypes. The module eigengenes (MEs) were defined as the significant components of the principal component analysis (PCA) for each gene module, where the expression level of every gene could be grouped to a distinct feature. We used a log10 transformation of the *p* value (GS = lg*p*) for the linear regression of correlations between clinical phenotypes and gene expression. Module significance (MS) was utilized to represent the correlation between clinical traits and gene expression calculated using the average GS in the module. Highly similar modules were merged as cut-off of <0.25, which could help cluster the key genes. The thresholds for the screening of key module genes were set as cor.gene GS > 0.2 and cor.gene MM > 0.8.

### 2.3. Functional and Pathway Enrichment Analysis

The R package “clusterProfiler” [15] was applied to assess the enriched biological pathways of the module genes in accordance with the Kyoto Encyclopedia of Genes and Genomes (KEGG) and Gene Ontology (GO). Single-sample gene set enrichment analysis (ssGSEA) was conducted to investigate pathways enriched in the DTL high- and low-expression subgroups. Briefly, ssGSEA transforms a single sample's gene expression profile to a gene set enrichment profile and calculates a separate enrichment score for each pairing of the sample and gene set, independent of phenotype labeling. And a gene set's enrichment score represents the activity level of the biological process in which the gene set's members are coordinately up- or downregulated. *h.all.v7.4.symbols.gmt* was chosen as the gene set database. The msigdbr, GSVA, and ggplot2 packages in R software were utilized for analysis and visualization. The pathways were considered significantly enriched with the following criteria: nominal *p* value < 0.05.

### 2.4. Human Bladder Tissue Samples

Paired BCa specimens and adjacent normal samples were generated from patients who underwent surgery at Zhongnan Hospital of Wuhan University. All patients understood and signed the informed consent. The fresh tumors and paracancerous tissues were immediately immersed in liquid nitrogen for subsequent experiments. The pathological diagnosis of each sample was independently confirmed by two pathologists. This study was approved by the Ethics Committee of Zhongnan Hospital of Wuhan University (approval number 20200507), and the utilization of the patients' information and specimens met the standards. All patients had signed informed consent before the study.

### 2.5. Cell Lines and Cell Culture

Human BCa cell lines (T24, UMUC3) were purchased from the Stem Cell Bank, Chinese Academy of Sciences in Shanghai, China, and verified by the China Centre for Type Culture Collection in Wuhan, China. T24 cells were cultured in RPMI-1640 medium (Gibco; Thermo Fisher Scientific, USA), and UMUC3 cells were cultured in MEM (Gibco; Thermo Fisher Scientific, USA). All cell cultures were supplemented with 10% foetal bovine serum (FBS, Gibco; Thermo Fisher Scientific, USA) and cultured at 37°C in a humidified incubator containing 5% CO_2_.

### 2.6. Tissue Microarray (TMA) and Immunohistochemical (IHC) Analysis

BCa TMA was purchased from Shanghai Outdo Biotech (Shanghai, China). TMA contained 56 bladder cancer specimens and 10 paracancerous tissues (HBlaU066Su01). The clinicopathologic and follow-up data of patients was obtained from http://www.superchip.com.cn/biology/tissue.html. IHC of BCa TMA was performed as described previously [16]. The BCa TMA was photographed with the Olympus BX53 biomicroscope. Each section was independently estimated by two pathologists. The IHC staining results were calculated by multiplying the staining intensity (strong = 3, moderate = 2, weak = 1, and negative = 0) with the percentage of immunoreactive cells (81-100% = 4, 51-80% = 3, 11-50% = 2, 1-10% = 1, and 0% = 0). The final scores were defined as strongly positive (3+; 9-12), moderately positive (2+; 6-8), weakly positive (1+; 2-4), and negative (0; 0-1).

### 2.7. RNA Inference, Retroviral Infection, and Cell Transfection

Small interfering RNAs targeting DTL (the sense sequence of DTL-siRNA was 5′-GCUAAUUGCACAGACGAUATT-3′, the antisense sequence of DTL-siRNA was 5′-UAUCGUCUGUGCAAUUAGCTT-3′, the negative control sense sequence was 5′-UUCUCCGAACGUGUCACGUTT-3′, and the antisense sequence was 5′-UAUCGUCUGUGCAAUUAGCTT-3′) were obtained from Genomeditech (Shanghai, China). Lentivirus short hairpin RNA of DTL was also constructed and packaged by Genomeditech (Shanghai, China). Lipofectamine 3000 (Invitrogen, Carlsbad, CA, USA) was utilized for cell transfection in accordance with the manufacturer's instructions.

### 2.8. RNA Extraction, Reverse Transcription, and Quantitative Real-Time PCR (qRT-PCR)

The RNeasy plus mini kits (Qiagen, Germany) were employed to extract total RNA from BCa cells and clinical specimens following the manufacturer's instructions. Subsequently, NanoDrop instrument (Implen, Germany) was utilized to evaluate the quality of the extracted RNA. Then, the RNA functioned as a template for cDNA synthesization using the ReverTra Ace qPCR RT Kit (Toyobo, Japan). Finally, cDNA forward and reverse primers and iQTM SYBR ® Green Supermix (Bio-Rad) were mixed, and the qRT-PCR was performed. The specific primer sequences were as follows: DTL-F: 5′-TGGTCTTCACAATACCCTCTTCA-3′, and DTL-R: 5′-CTTCATTGGCAACTGCTAGTACA-3′, and GAPDH-F: 5′- GGAGCGAGATCCCTCCAAAAT-3′, and GAPDH-F: 5′-GGCTGTTGTCATACTTCTCATGG-3′. The expression of the targeted genes was normalized to GAPDH.

### 2.9. Cell Proliferation Assay

The methyl thiazolyl tetrazolium (MTT) assay and clone formation assay were implemented to detect cell proliferation. After 48 hours of transfection, UMUC3 and T24 cells were collected and counted under the microscope. For the MTT assay, 3 × 10^3^ cells were seeded in 96-well culture plates with 6 repeated wells for growth. Then, take out a 96-well plate every 24 hours for five consecutive days, add 20 *μ*l MTT reagent (5 mg/ml, Sigma-Aldrich) into each well, and put it in the incubator for four hours and measure the absorbance of each well at 490 nm. For the clone formation assay, cells were seeded into 6-well plates at a density of 1 × 10^3^ cells per well. After 14-day incubation, cells were fixed with 4% paraformaldehyde and visualized by 0.5% crystal violet staining.

### 2.10. Flow Cytometry Analysis

48 hours after cell transfection, UMUC3 and T24 cells were sequentially digested, centrifuged, and washed twice with PBS. Under dark condition, 1× DNA staining solution (Multisciences, China), containing permeabilization solution and propidium iodide, was utilized to resuspend the cells for 30-minute incubation at room temperature, and then, flow cytometry (cat. no. FC500; Beckman Coulter, USA) was applied to analyze the distribution of the cell cycle.

### 2.11. Cell Migration and Invasion Assays

A wound healing assay and transwell chambers (Corning, USA) were performed to assess cell migration and invasion capability. For the transwell migration assay, 1 × 10^4^ cells were resuspended in 200 *μ*l serum-free medium and seeded in the upper chamber of the transwell system. In the meantime, 600 *μ*l complete nutrient medium was added to the lower chamber to induce cell migration. After 24 hours in the incubator, take out the transwell system and aspirated medium, wipe off the unmigrated cells in the upper chamber, add 4% PFA to fix the cells for 1 hour, and stain the migrated cells with 0.1% crystal violet. For the transwell invasion assay, firstly, ECM Matrix gel solution (Sigma-Aldrich, St. Louis, MO, USA) was seeded in transwell chambers, solidifying at 37°C; the subsequent incubation and staining procedures were the same as the migration assays. Finally, all the samples were observed under a 200x microscope (Olympus) to capture images from five randomly selected fields. For the wound healing assay, the transfected UMUC3 and T24 cells continue to be cultured in six-well plates to 80% density; then, use a 200 ml pipette tip to vertically scratch the cells, wash away the scratched cells with PBS, and add 1 ml culture medium to continue to cultivate for 24 hours. Finally, observe and calculate the average wound gap between wound edges.

### 2.12. Immunofluorescence Assay

Twelve-millimetre coverslips with T24 cells were washed three times with ice-cold PBS and fixed with 4% PFA for 15 min. The cells were then treated with a 0.1% Triton X-100 solution and blocked with normal goat serum for 30 min at room temperature. The fixed cells were incubated with the indicated antibodies at the proper dilution for 2 h at room temperature, washed three times with PBS, and incubated with secondary antibodies for 1 h. Nuclei were visualized by incubating with DAPI (2 *μ*g/ml) for 10 min at room temperature, and slides were analyzed by using a biological microscope (Olympus BX53).

### 2.13. Western Blotting (WB) Analysis

Cells were lysed with the RIPA extraction reagent (Beyotime, China) supplemented with protease inhibitors (Sigma-Aldrich, USA). Equal amounts of total protein were separated using 10-12.5% sodium dodecyl sulfate polyacrylamide gel electrophoresis and transferred to 0.45 *μ*m PVDF membrane (Millipore, USA). The membranes were blocked with 5% nonfat dry milk in TBST for 2 hours at room temperature and then incubated with specific primary antibodies overnight at 4°C followed by incubation with HRP-conjugated secondary antibodies at 37°C for 2 h at room temperature. Immune response bands were exposed via a Bio-Rad ChemiDoc XRS+ Imaging System (Bio-Rad, USA). Primary antibodies were DTL (Abcam, cat# ab72264), P21(CST, cat# 2947), CDK1(Abcam, cat# ab133327), CDK2 (Abcam, cat# ab32147), CDK4 (CST, cat# 12709), cyclinB1 (CST, cat# 12231S), CDT1(Abcam, cat# ab202067), SETD8(Abcam, cat# ab230683), E-cadherin (CST, cat# 3195S,), N-cadherin (CST, cat# 13116S), AKT (CST, cat# 4691L), p-AKT (CST, cat# 4060L,), GSK-3*β* (CST, cat# 12456S), p-GSK-3*β* (CST, cat# 5558S), mTOR (Abcam, cat# ab32028), p-mTOR (Abcam, cat# ab109268), and GAPDH (Santa Cruz, cat# sc-365062) antibodies.

### 2.14. In Vivo Tumorigenesis Assay

Three-week-old male BALB/c nude mice were obtained from Beijing HFK Bioscience Co., Ltd. in Beijing, China, and the mice were allowed to adapt for a week without any treatment in the laboratory animal facility of Zhongnan Hospital of Wuhan University. At the beginning, the mice were randomly divided into the experimental group and the control group. For the construction of the xenograft model, 1 × 10^6^ UMUC3 cells were intravenously injected to the right dorsal flank of each mouse. Tumor sizes were measured every five days until the end of the experiment. 25 days later, the mice were injected intraperitoneally with pentobarbital at the dose of 100 mg/kg and then euthanized by cervical dislocation, and the tumors were removed. The experiments were implemented in accordance with the protocols approved by the ethic committee of Zhongnan Hospital of Wuhan University.

### 2.15. Statistical Analysis

R software (version 3.5.2) was utilized for statistical processing and figure formatting. Two-sided *p* < 0.05 was considered statistically significant. Categorical variables were expressed as a component ratio or rate, and their comparison between groups was conducted by the chi-square test or continuity correction of the chi-square test. Group comparisons were conducted utilizing Student's *t*-test for continuous variables. The receiver operating characteristic (ROC) curve and the area under the curve (AUC) were applied to evaluate the diagnosis accuracy with the package “pROC.” Then, the Kaplan-Meier survival method was adopted to assess the survival prognosis, followed by the log-rank test for comparing differences between DTL high- and low-expression groups. Univariate and multivariate Cox proportional hazard models were applied to identify independent prognostic factors influencing the survival rate.

## 3. Results

### 3.1. Integrative Bioinformatics Analysis of the DTL Gene in BCa Microarray

**Figure 1:**
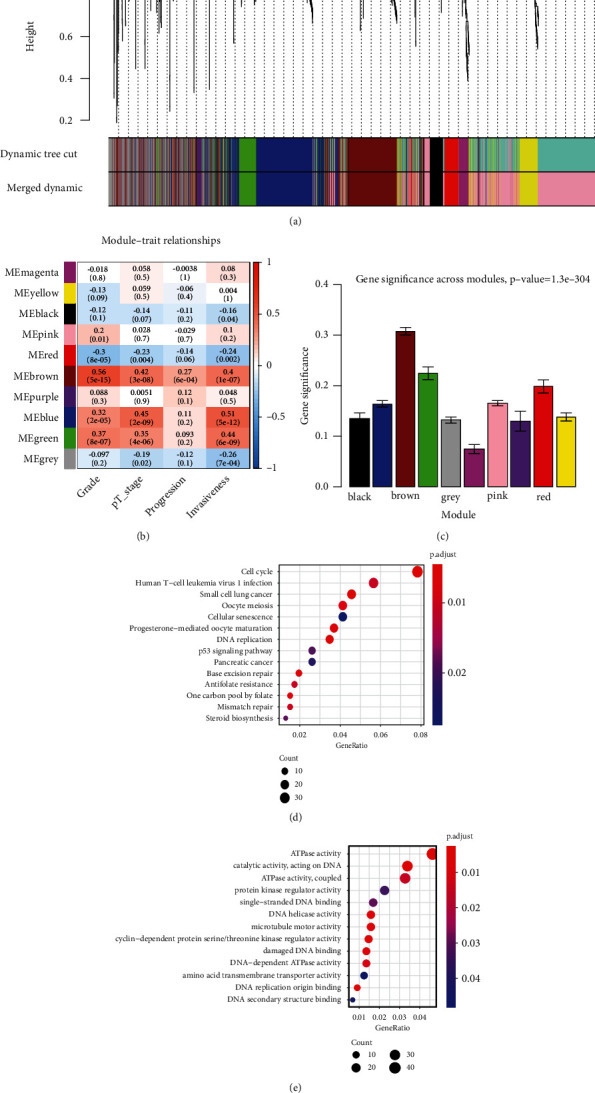
Weighted gene coexpression network analysis identified key modules associated with BCa progression. (a) Dendrogram of all selected genes clustered based on a dissimilarity measure (1-TOM). (b) Heatmap of the correlation between module eigengenes and different clinical information of BCa. (c) Distribution of the average gene significance and errors in the modules associated with tumor stage and grade of BCa. (d) Bubble chart showing Gene Ontology and KEGG pathway enrichment analysis based on all genes in the brown module. The *x*-axis represents -log10 (*p* value), and the *y*-axis represents the significantly enriched pathways.

**Figure 2:**
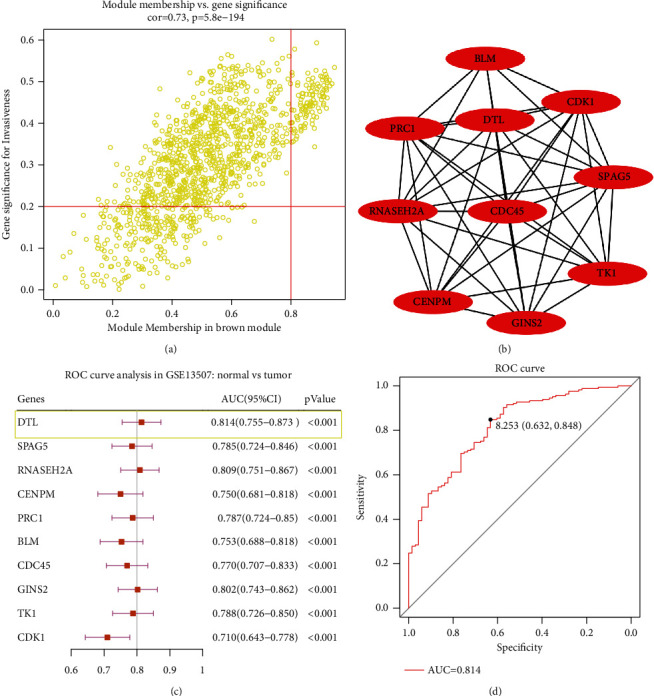
DTL was a key gene and an excellent diagnostic biomarker in BCa. (a) The scatter plot of all genes in the brown module; those circles located in the upper right indicate the key genes in these modules. (b) The protein-protein interaction network identified top 10 hub genes based on the STRING database. (c) ROC curve analysis of the selected top 10 hub genes in GSE13507. (d) DTL diagnosis ROC curve analysis in GSE13507.

The GSE13507 dataset was applied to perform bioinformatics analysis. After normalizing all genes in the matrix in GSE13507, we selected the top 25% of the genes with the largest variance in GSE13507 for WGCNA construction. After sample clustering analysis, 164 bladder cancer samples with complete clinical data were incorporated into coexpression analysis (Supplementary Figure 1A-B). Power of *β* = 7 (scale-free *R*^2^ = 0.91) was selected to ensure a scale-free network (Supplementary Figure 2A-D). A total of 10 modules were identified after using average linkage clustering (Figure 1(a)); then, the relevance was identified between each module and the clinical features with great biological significance. The brown module was dramatically associated with clinical features of pathological progression (Figures 1(b) and 1(c)). Pathway enrichment analysis demonstrated that all genes were enriched in cell cycle- and DNA replication-associated pathways. In accordance with the criteria that cor.gene module membership > 0.8 and cor.gene trait significance > 0.2, 114 genes with the high connectivity in the brown module were screened as hub genes (Supplementary Table 1). Then, we also implemented a protein-protein interaction (PPI) network analysis for all hub genes based on the STRING database (https://string-db.org/), and DTL was indicated as one of the top 10 hub genes (Figures 2(a) and 2(b)). Further, ROC curve analysis was performed to evaluate the diagnostic value of top 10 hub genes in BCa. The result indicated that the expression of DTL performed excellent diagnostic efficiency for tumor and normal tissues (Figures 2(c) and 2(d)). So DTL may be an excellent biomarker for BCa.

### 3.2. DTL Was Overexpressed in BCa and Correlated with Clinicopathologic Characteristics of BCa Patients

**Figure 3:**
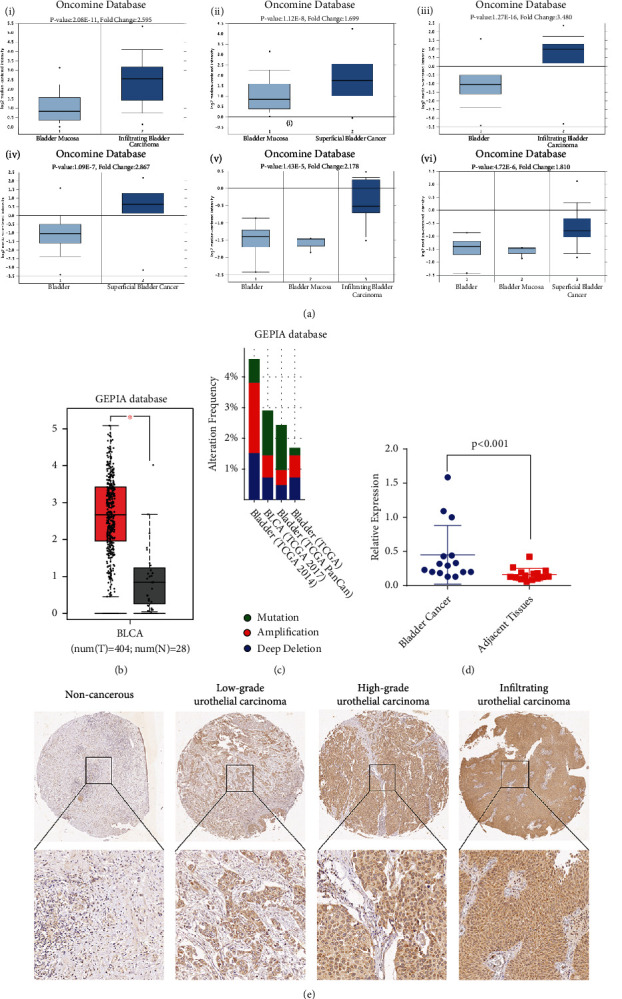
DTL is overexpressed in bladder cancer tissues compared with the adjacent normal tissues. (a) DTL expression analysis in the Oncomine database. (b) DTL expression analysis in the GEPIA database. (c) Alteration frequency of DTL in BCa. (d) qRT-PCR result of 15 paired BCa tumor tissues and adjacent tissues. (e) IHC staining of DTL in bladder cancer tissues. Images are presented at ×100 (upper) and ×400 (lower) magnification.

DTL mRNA expression in BCa and corresponding normal tissues was explored using the Oncomine database. The results demonstrated that DTL mRNA was dramatically increased in BCa, compared with normal bladder mucosa and paracancerous bladder tissues (Figure 3(a), *p* < 0.05). Moreover, we validated it in the GEPIA database. The result was consistent with the Oncomine database (Figure 3(b)), and DTL gene mutation and amplification were present in most BCa samples (Figure 3(c)). Then, we used 15 pairs of BCa tissues and paracancerous bladder tissues in Zhongnan Hospital for validation. The qRT-PCR results also demonstrated that DTL was overexpressed in BCa tissues (Figure 3(d)). Moreover, immunohistochemical staining of BCa tissues was performed to assess the DTL protein expression. We found that protein expression level of DTL was higher in BCa tumor tissues than the adjacent normal tissues. With tumor progression, protein expression level of DTL was further increased (Figure 3(e)). Then, we evaluated the correlation between DTL and clinicopathological characteristics, as Table 1 shows that DTL expression was significantly correlated with age, gender, invasiveness, T stage, lymphatic metastasis, and grade.

### 3.3. DTL Could Be an Independent Prognostic Biomarker for BCa

**Figure 4:**
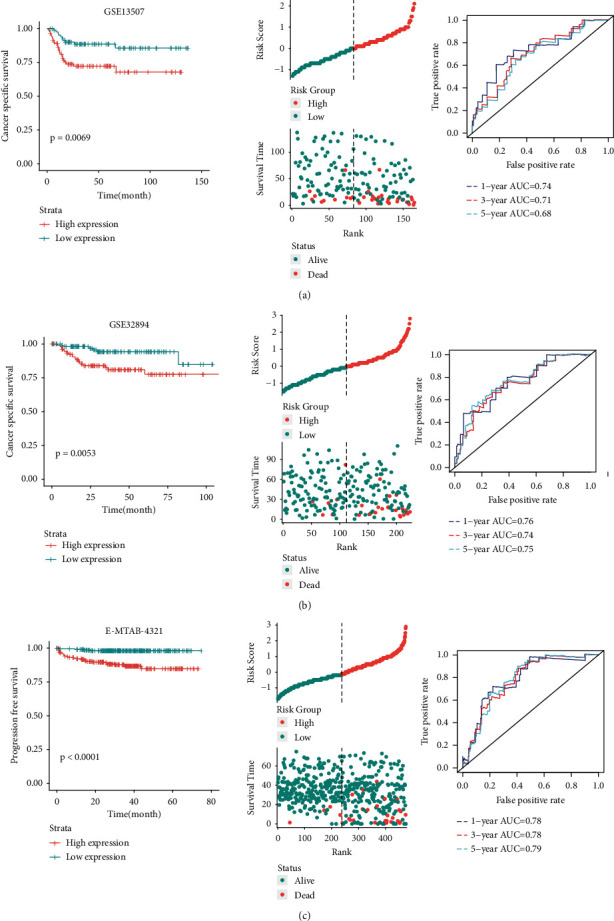
Prognostic analysis of DTL in three independent datasets. (a) GSE13507 dataset. (b) GSE32894 dataset. (c) E-MTAB-4321 dataset. Left panels indicated Kaplan-Meier survival curves in three datasets, and patients with DTL high expression represented dramatically unfavorable survival than those with DTL low expression. Middle panels indicated the distributions of all the patients' survival status in the DTL low-expression group and high-expression group. Right panels indicated the time-dependent ROC curves for survival prediction in three datasets.

**Figure 5:**
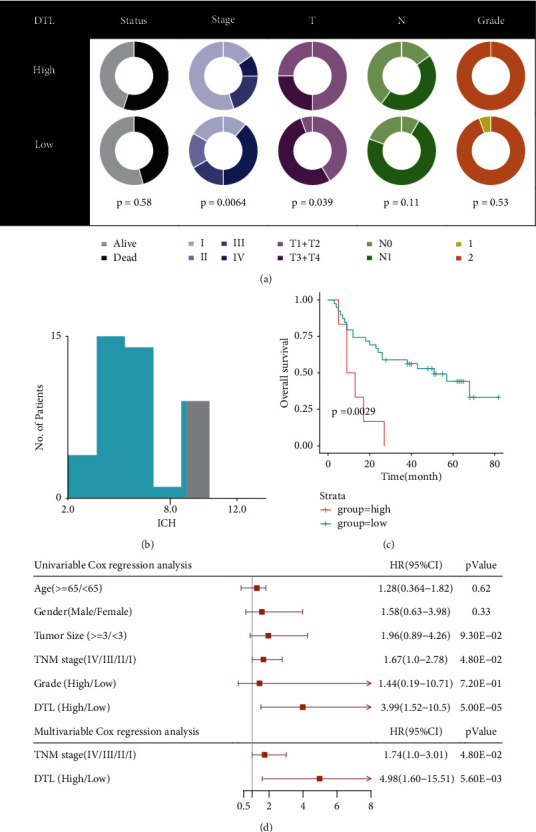
Clinical relevance and prognostic analysis of DTL in BCa tissue microarray. (a) DTL expression was associated with the tumor stage of BCa. (b) Defining the optimum cut-off value using X-tile software. (c) High expression of DTL was associated with unfavorable prognosis. (d) Cox regression analysis represented that DTL was an independent prognostic factor for BCa.

We performed Kaplan-Meier analysis to investigate whether DTL expression could be used as a potential specific prognosis marker for BCa in the GSE13507 dataset. As shown in Figure 4(a), high DTL expression was significantly associated with poor cancer-specific survival (CSS) (*p* = 0.0069). Another two datasets, GSE32894 and E-MTAB-4321, were used to validate the results, and the results indicated that DTL was also associated with poor CSS and progression-free survival (PFS) (Figures 4(b) and 4(c)). A tissue microarray (TMA) was used to verify the results of gene expression profiles. Higher DTL expression was significantly associated with the tumor stage (Figure 5(a)). We utilized X-tile software to define the optimum cut-off value (Figure 5(b)). All patients were divided into high-expression and low-expression groups based on the cut-off value, and patients in the high-expression group showed statistically significantly poorer prognoses than did patients in the low-expression group (*p* = 0.0029, Figure 5(c)). Cox regression analysis demonstrated that DTL was an independent predictor for poor overall survival (Figure 5(d)). These results emphasized that DTL is overexpressed in BCa tumors and is an independent prognostic factor for poor survival.

### 3.4. Functional and Pathway Enrichment Analysis

**Figure 6:**
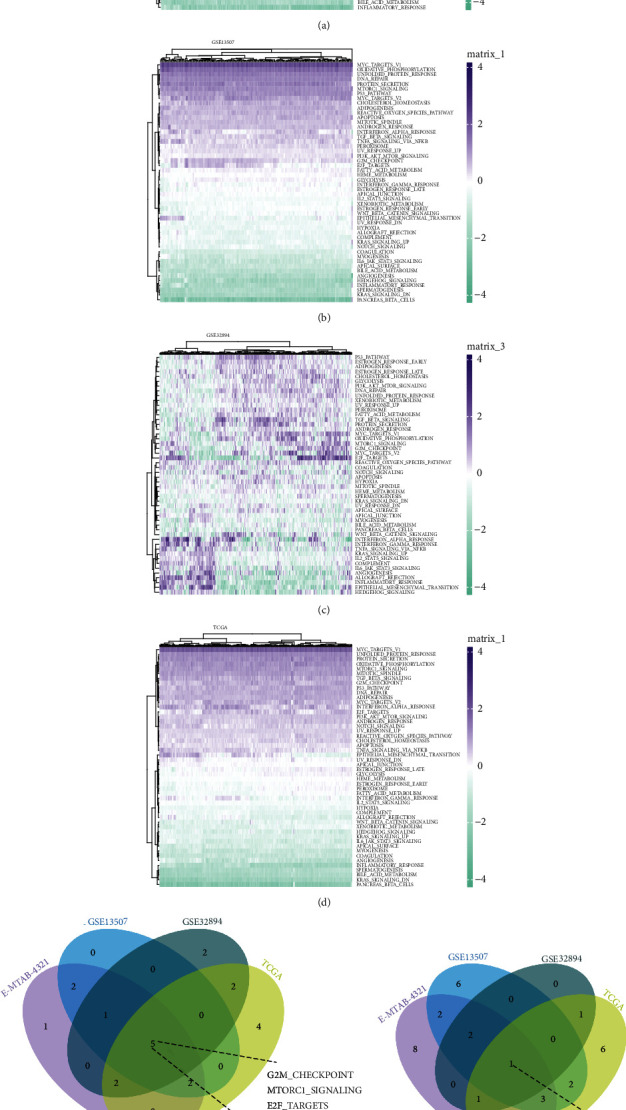
ssGSEA enrichment pathway-based DTL expression. (a–d) ssGSEA enrichment pathways in E-MTAB-4321, GSE13507, GSE32894, and TCGA datasets, respectively. (e) Venn diagram of the activated pathways in the four datasets. (f) Venn diagram of the suppressed pathways in the four datasets.

Moreover, ssGSEA analysis was performed in E-MTAB-4321, GSE13507, GSE32894, and TCGA-BCa datasets, respectively (Figures 6(a)–6(d)), and a summary of the GSEA results in each dataset is shown in Supplementary Tables 2–5. Five activated gene sets, G2M checkpoint, E2F targets, mTORC1 signing, MYC targets V1, and UV response UP, were enriched in the DTL high-expression group; however, one suppressed gene set, adipogenesis, was enriched in the DTL high-expression group (Figures 6(e) and 6(f)). These results indicated that DTL may promote tumorigenesis and progression by regulating cell cycle- and DNA replication-associated pathways.

### 3.5. Depletion of DTL Inhibited the Proliferation and Colony Formation of BCa Cells

**Figure 7:**
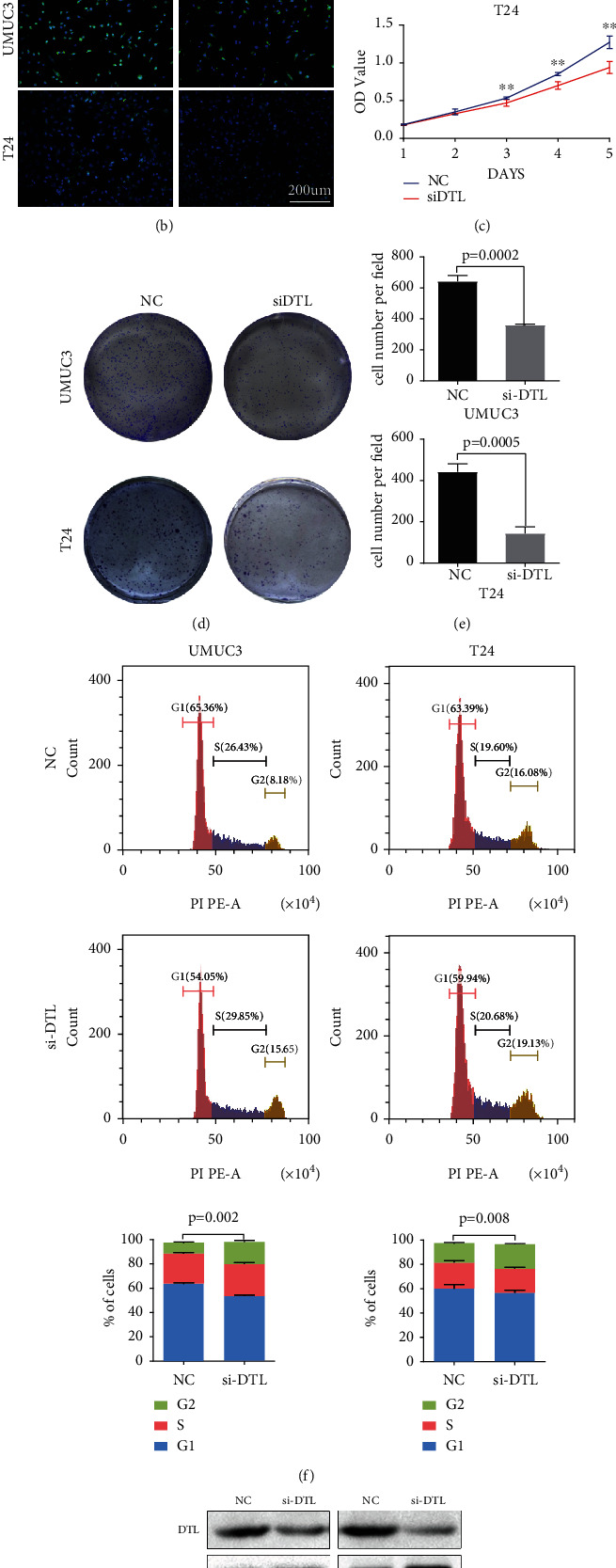
Depletion of DTL suppresses BCa cell proliferation and restrains G2 cell cycle transition. (a) qRT-PCR analysis assessed knockdown efficiency of three DTL siRNAs in UMUC3 and T24 cells, and siRNA1 represents the highest knockdown efficiency. Therefore, siRNA1 was chosen to be used in subsequent experiments. (b) Immunofluorescence assessed knockdown efficiency of siRNA1 in UMUC3 and T24 cells. (c) MTT assays in UMUC3 and T24 cells represented cell viability after depletion of DTL. (d) Clone formation assay represented cell proliferation viability after depletion of DTL, and (e) the clone formation cell counts were statistically analyzed. (f) Flow cytometry represented the alteration of the cell cycle after depletion of DTL in UMUC3 and T24 cells, and statistical significance was assessed using two-tailed *t*-tests. (g) Western blotting analysis represented the alteration of cycle-associated protein. ^∗^*p* < 0.01, ^∗∗^*p* < 0.001.

Next, we explored the role of DTL in regulating BCa cell proliferation viability in vitro. We depleted the DTL gene in T24 and UMUC3 (Figures 7(a) and 7(b)), and MTT results demonstrated that depletion of DTL significantly attenuated cell proliferation viability (Figure 7(c)). The results of the clone formation assay revealed that DTL depletion dramatically decreased the clone formation capability (Figures 7(d) and 7(e)). Flow cytometry results implied that depletion of DTL significantly decreased cell proliferation and increased the population in G2 phases, indicating that DTL may regulate G2 to M transition in BCa cells. Moreover, the western blotting results indicated that key proteins of the cell cycle including CDK1, CDK2, CDK4, P21, cyclinB1, CDT1, and SETD8 were attenuated (Figure 7(g)).

### 3.6. Depletion of DTL Inhibited the Migration and Invasion of BCa Cells

**Figure 8:**
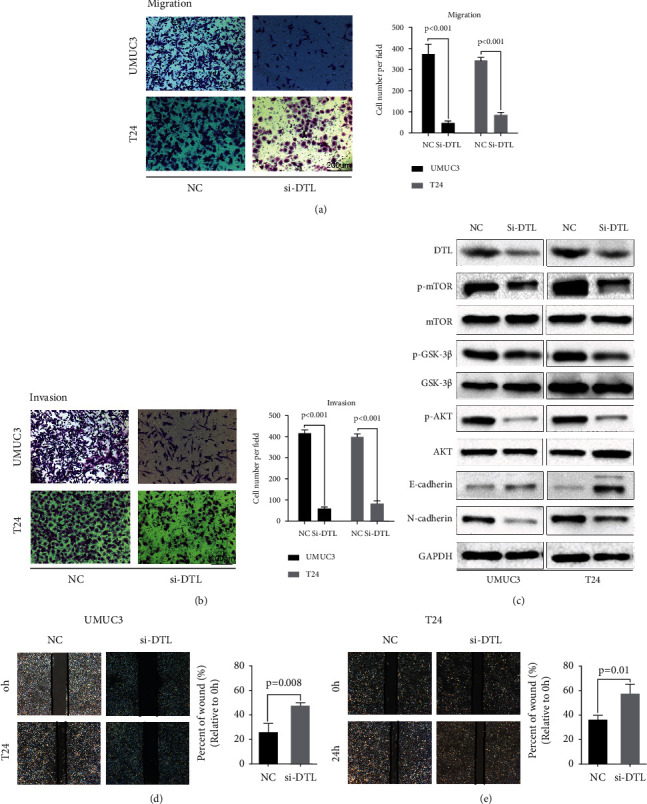
Depletion of DTL suppresses BCa cell migration and invasion, attenuates the EMT of BCa cells, and inhibits the AKT/mTOR pathway. (a, b) Transwell migration and invasion assays in UMUC3 and T24 cells represented cell migration and invasion viability after depletion of DTL, and the migrated or invaded cell counts were statistically analyzed. (c) Western blotting analysis revealed the expression alterations of EMT markers and key proteins of the AKT/mTOR pathway after depletion of DTL. GAPDH was used as an internal control. (d, e) Wound healing assay represented cell migration viability after depletion of DTL, and the migrated cell counts were statistically analyzed. Statistical significance was assessed using two-tailed *t*-tests.

Further, the altered migration and invasion viability of UMUC3 and T24 cells was detected via the transwell assay and wound healing assay. The results revealed that the migration and invasion ability of BCa cells in the si-DTL group was attenuated, compared with that in the control group (Figures 8(a) and 8(b)). EMT is regarded as a pathological process leading to tumor progression, EMT enables tumor cells to invade and metastasize, and EMT marker (E-cadherin and N-cadherin) expressions were detected via western blotting. The results showed that E-cadherin level was upregulated and N-cadherin levels were downregulated, when DTL was knocked down by siRNAs (Figure 8(c)). Thus, DTL promotes BCa cell migration and invasion. We further investigate how DTL upregulates the aggressive abilities of BCa cells. As DTL significantly activates MTORC1 signaling by ssGSEA analysis, western blotting showed that the mTOR level is attenuated when DTL was deleted; moreover, upstream markers of mTOR, AKT, and gsk3*β* were attenuated (Figure 8(c)). In conclusion, DTL is associated with AKT/gsk3*β*/mTOR activation.

### 3.7. Reduction of DTL Suppresses BCa Cell Growth In Vivo

**Figure 9:**
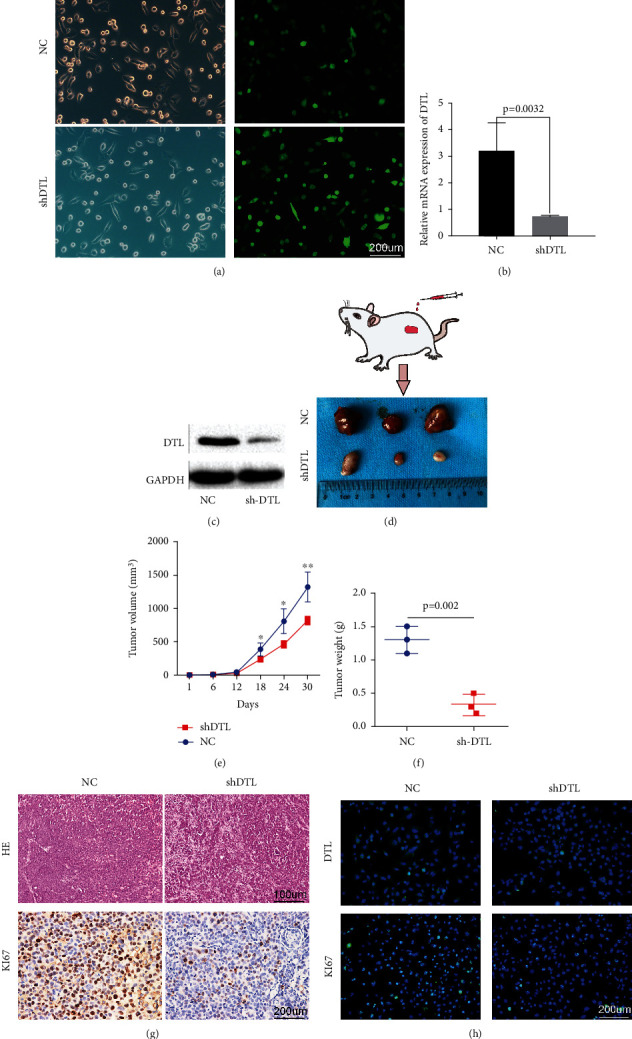
Reduction of DTL suppresses BCa growth in vivo. (a) The green fluorescence of UMUC3 stable cells. (b, c) qRT-PCR and western blotting analysis verified DTL depletion efficiency in UMUC3 stable cells. (d) Xenograft mouse models were established by subcutaneously injecting LV-NC cells or LV-shDTL cells and monitored continuously for 5 weeks; then, the mice were sacrificed and the tumors were dissected. (e, f) Statistical analysis of tumor volume and weight in two groups. (g) HE staining assessed the nucleus atypia of tumors, and IHC staining detected the expression of Ki67. (h) Immunofluorescence staining detected the expression of DTL and Ki67. ^∗^*p* < 0.01, ^∗∗^*p* < 0.001.

Then, we further investigated the role of DTL in tumor growth in the xenograft mouse model. Firstly, we obtained UMUC3 cell lines with stable depletion of DTL by lentivirus-based shRNA (Figure 9(a)), and stable depletion efficiency of DTL was detected by qRT-PCR and western blotting (Figures 9(b) and 9(c)). Then, UMUC3 LV-NC cells and UMUC3 LV-shDTL cells were separately injected into BALB/c nude mice to construct the xenograft mouse model. As presented in Figures 9(d) and 9(e), the shDTL group had a slower growth of tumors compared with the NC group. Accordingly, average weight of tumors of the shDTL group was dramatically lighter than that of the NC group (Figure 9(f)). Moreover, the dissected tumors were assessed by HE, IHC, and immunofluorescence staining. The HE staining demonstrated that the shDTL group has a lower degree of nucleus atypia, and the IHC staining revealed that the positive staining of Ki67 was lower in the shDTL group than in the NC group (Figure 9(g)). Accordingly, immunofluorescence analysis of the xenograft tumors showed that the expression levels of DTL and Ki67 in the shDTL group were decreased compared with those in the NC group (Figure 9(h)). These results indicated that depletion of DTL decelerated BCa growth in vivo.

## 4. Discussion

In this study, our data revealed that DTL was one of the hub genes and an excellent diagnostic biomarker in BCa; it was remarkably upregulated in BCa tumor tissues, which was associated with higher TNM stage and worse survival. Increased DTL was also identified as an independent prognosis factor to predict the poor outcomes of patients with BCa. All these results indicate that DTL could be used as a potential biomarker for BCa.

A cancer hub gene is a gene that plays a vital role in the biological process. It often regulates other genes in related pathways as a key gene. Therefore, hub genes are often important targets and research hotspots, an interaction network can be constructed through coexpression or protein interaction, and then key genes can be screened according to the network topology. In this study, we performed WGCNA that we have identified the key modules for the effect of BCa progression, including 114 genes. Through pathway enrichment analysis, we found that these genes are mainly enriched in pathways associated with the cell cycle and DNA replication. We further identified 10 hub genes through STRING network analysis; DTL, as one of the hub genes, was identified as a potential biomarker through ROC curve analysis. Further analysis revealed that DTL was significantly overexpressed in BCa. Survival analysis showed that DTL was significantly negatively correlated with BCa patients, and patients with high DTL expression showed significantly poor prognosis.

DTL, also known as DNA replication factor 2 (CDT2) or retinoic acid-regulated nuclear matrix-associated protein (RAMP), is a member of DCAF family genes that encode substrate receptor proteins for Cullin-RING E3 ubiquitin ligases. Together with CUL4A, DDB1, and RBX1 proteins, it forms the E3 complex CRL4A. The potential roles of DTL have been explored in human cancers, for example, ovarian cancer, melanoma, colon cancer, breast cancer, and hepatocellular carcinoma [17–21]. Cui et al. reported that DTL which is upregulated in DTL is aberrantly upregulated in breast cancer. Both the overall survival and the recurrence-free survival of patients with high expression of DTL are shorter than those with low expression of DTL. In vitro experiments demonstrated that DTL interacts with PDCD4 and ubiquitinates PDCD4 to promote the migration, invasion, and proliferation of breast cancer cells [21]. Studies have also reported that DTL is associated with poor overall survival and disease-free survival in cutaneous melanoma [22, 23], and inhibition of DTL induces DNA rereplication and senescence by MLN4924 and suppresses melanoma in vitro and in vivo [24]. Li et al.'s and Kobayashi et al.'s researches found DTL is an oncogene in gastric cancer, and overexpression of DTL is related to poor survival in gastric cancer [16, 25]. However, the characteristics and biological functions of DTL in BCa have not been reported yet. Here, our data proved that DTL is overexpressed in BCa and is a potential biomarker for the diagnosis and prognosis of BCa. Moreover, DTL is associated with the BCa proliferation, migration, and invasion.

As a substrate recognition factor of E3 ubiquitin ligase, DTL can recognize and degrade multiple substrates to regulate a variety of cell biological functions, such as DNA replication, posttranscriptional modification, apoptosis, and DNA repair [26–28]. Through ssGSEA enrichment analysis, we found that DTL activates pathways associated with DNA replication and cell cycle. Previous research showed that DTL mediates destruction of cell cycle-related proteins, SETD8, CDT1, and P21, to prevent premature chromatin compaction in the S phase [29, 30] and is essential for the early G2/M checkpoint [31]. Our data is consistent with the report, and depletion of DTL can increase the steady-state level of SETD8, CDT1, and P21 and induce cell cycle G2/M phase arrest.

EMT is a histopathological process in which tumor cells gradually lose the characteristics of epithelial cells and acquire the characteristics of mesenchymal cells. In this process, the cells gradually lose their cell polarity, their connection with the basement membrane, and their morphology. The transformation to a fusiform, the gradual loss of adhesion, and higher migration and invasion capabilities are the important characteristics of tumor metastasis. Through the detection of the EMT marker, we found that depletion of DTL attenuates the expression levels of N-cadherin and increased the expression level of E-cadherin, indicating that DTL promotes the process of EMT to induce the migration and invasion ability of cancer cells. Evidence indicates that the AKT/mTOR signaling axis significantly regulates the malignant phenotypes of cancer cells, such as proliferation, metastasis, and EMT [32–34], and is related to the chemoresistance and radioresistance in various tumors [35, 36]. This signaling pathway also plays an important role in carcinogenesis and progression of BCa [37–39]. In our study, when DTL was knocked down, we observed that the phosphorylated protein levels in the AKT/mTOR signaling pathway were significantly reduced, accompanied by upregulated E-cadherin and decreased N-cadherin. Accordingly, DTL may promote BCa progression though activation of the AKT/mTOR signaling pathway.

However, there are still some limitations in our researches. Firstly, our sample size was limited, and larger clinical sample size is needed to verify the correlation between DTL and the clinicopathology and prognosis of BCa. Secondly, it is necessary to demonstrate the specific mechanism for further experiments by which DTL induces the activation of the AKT/mTOR pathway and EMT. Therefore, our findings provide a foundation for further research on the specific mechanisms of DTL promoting BCa progression.

## 5. Conclusions

In summary, based on integrated bioinformatics analysis and experiment validation, we found that DTL was overexpressed in BCa. Increased DTL expression was correlated with malignant biological behavior and promoted BCa progression through the AKT/mTOR pathway. It may be used as a potential diagnosis and therapeutic target for BCa.

## Figures and Tables

**Table 1:** Association between the expression of DTL and clinicopathological characteristics.

Variable	Total patients	DTL expression		*p*
	No (%)	Low	High	
Age, mean ± SD (years)	65.2 ± 12	61.9 ± 12.5	68.5 ± 10.5	0.0003
Age				0.0004
<65	74 (44.8%)	49 (59.0%)	25 (30.5%)	
≥65	91 (55.2%)	34 (41.0%)	57 (69.5%)	
Gender				0.024
Male	135 (81.8%)	74 (89.2%)	61 (74.4%)	
Female	30 (18.2%)	9 (10.8%)	21 (25.6%)	
Invasiveness				0.005
Superficial	103 (62.4%)	61 (73.5%)	42 (51.2%)	
Invasive	62 (37.6%)	22 (26.5%)	40 (48.8%)	
Lymphatic metastasis				0.03
No	149 (90.3%)	79 (95.2%)	70 (85.4%)	
Yes	15 (9.1%)	3 (3.6%)	12 (14.6%)	
T stage				0.004
Ta	24 (14.5%)	19 (22.9%)	5 (6.1%)	
T1	80 (48.5%)	42 (50.6%)	38 (46.3%)	
T2	31 (18.8%)	13 (15.7%)	18 (22.0%)	
T3	19 (11.5%)	4 (4.8%)	15 (18.3%)	
T4	11 (6.7%)	5 (6.0%)	6 (7.3%)	
Grade				2.01*e* − 06
Low	105 (63.6%)	68 (81.9%)	37 (45.1%)	
High	60 (36.4%)	15 (18.1%)	45 (54.9%)	

## Data Availability

The datasets used and/or analyzed during the current study are available from the corresponding author on reasonable request.

## Abbreviations

AUC: Area under the curve
BCa: Bladder cancer
CDT2: DNA replication factor 2
DTL: Denticleless E3 ubiquitin protein ligase homolog
EMT: Epithelial mesenchymal transition
TCGA: The Cancer Genome Atlas
GEO: Gene Expression Omnibus
GO: Gene Ontology
GS: Gene significance
KEGG: Kyoto Encyclopedia of Genes and Genomes
MM: Module membership
qRT-PCR: Quantitative real-time PCR
RAMP: Retinoic acid-regulated nuclear matrix-associated protein
ROC: Receiver operating characteristic curve
ssGSEA: Single-sample gene set enrichment analysis
TMA: Tissue microarray
TOM: Topological overlap matrix
WGCNA: Weighted correlation network analysis.

## Supplementary Materials

Supplementary 1 Supplementary Figure 1: clustering dendrogram of BCa samples and the clinical traits. (A) BCa sample clustering in the GSE13507 dataset. (B) Dendrogram of BCa samples and the clinical trait heatmap. The red color represents tumor grade, tumor stage, tumor progression, and microvascular invasion. The color intensity represents higher stage, grade, and pathological progression. Supplementary Figure 2: determine soft thresholding power in WGCNA. (A) The scale-free fit index for various soft thresholding powers. (B) The mean connectivity for various soft thresholding powers. (C) Histogram of connectivity distribution (*β* = 7). (D) Checking the scale-free topology (*β* = 7).

Supplementary 2 Supplementary Table 1: hub genes in the brown module. Supplementary Table 2: ssGSEA enrichment analysis in E-MTAB-4321. Supplementary Table 3: ssGSEA enrichment analysis in GSE13507. Supplementary Table 4: ssGSEA enrichment analysis in GSE32894. Supplementary Table 5: ssGSEA enrichment analysis in TCGA-BLCA.
