# Supplementary material for: DTL Is a Prognostic Biomarker and Promotes Bladder Cancer Progression through Regulating the AKT/mTOR axis
Source: Oxid Med Cell Longev. 2022 Jan 21;2022:3369858. doi: 10.1155/2022/3369858 (PMC8799954; doi:10.1155/2022/3369858)
Supplement: Supplementary 1 — Supplementary Figure 1: clustering dendrogram of BCa samples and the clinical traits. (A) BCa sample clustering in the GSE13507 dataset. (B) Dendrogram of BCa samples and the clinical trait heatmap. The red color represents tumor grade, tumor stage, tumor progression, and microvascular invasion. The color intensity represents higher stage, grade, and pathological progression. Supplementary Figure 2: determine soft thresholding power in WGCNA. (A) The scale-free fit index for various soft thresholding powers. (B) The mean connectivity for various soft thresholding powers. (C) Histogram of connectivity distribution (β = 7). (D) Checking the scale-free topology (β = 7). [file 3369858.f1.docx]

Supplementary Figures


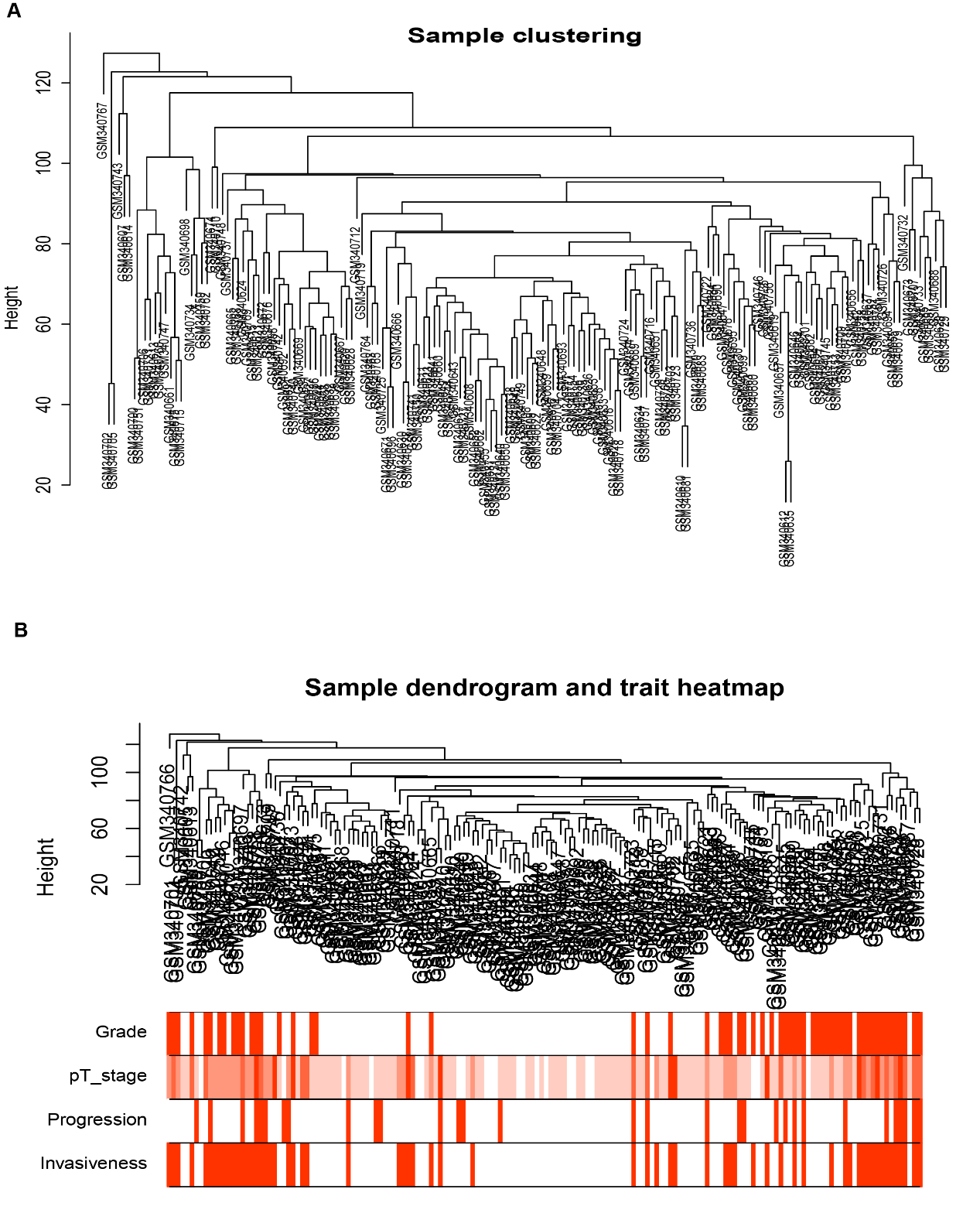


Figure S1. Clustering dendrogram of BCa samples and the clinical traits, (A) BCa sample clustering in GSE13507 dataset. (B) Dendrogram of BCa samples and the clinical traits heatmap. The red color represents tumor grade, tumor satge, tumor progression and microvascular invasion. The color intensity represents higher stage, grade and pathological progression.


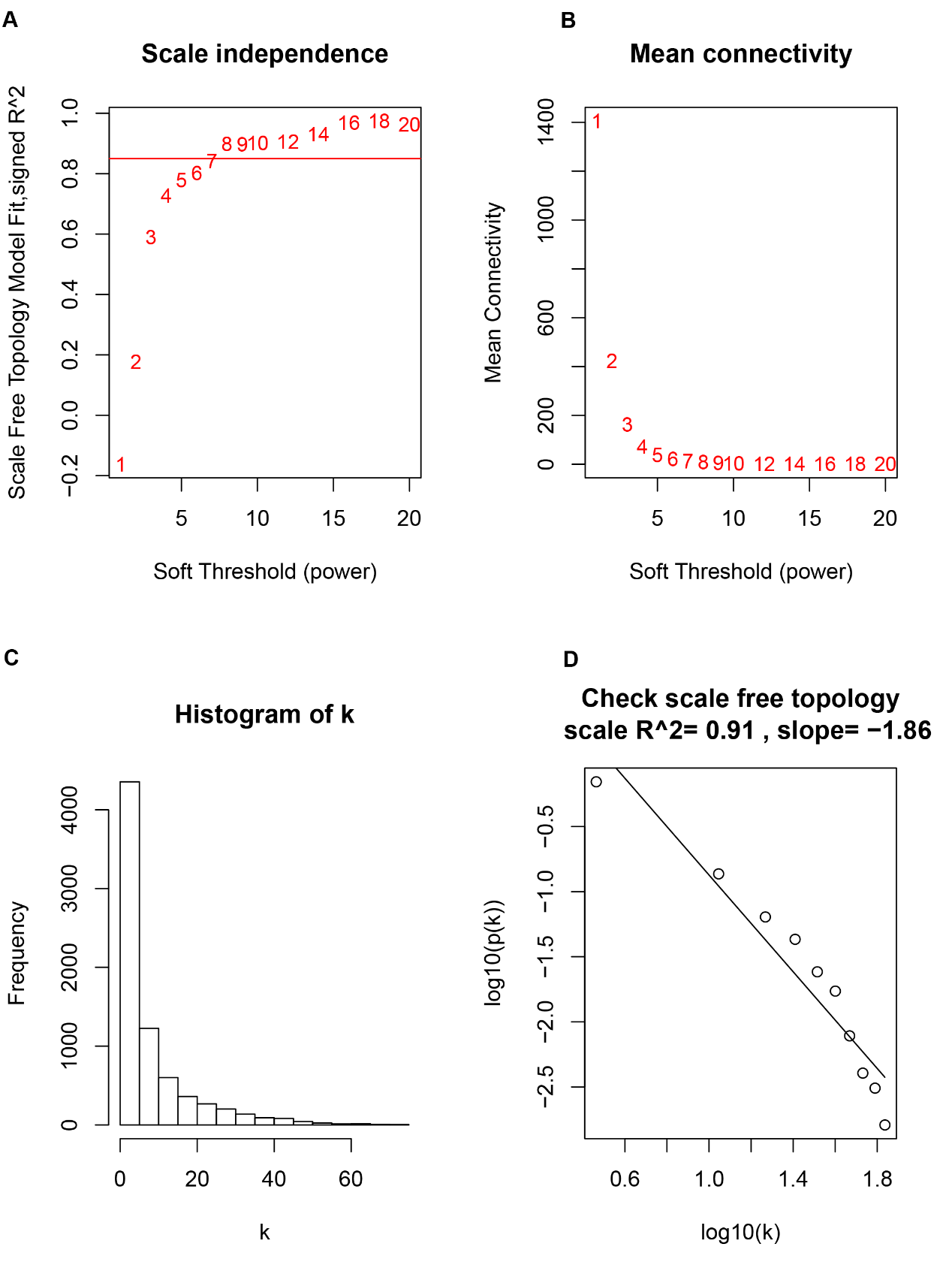


Figure S2. Determine soft-thresholding power in WGCNA. (A) The scale-free fit index for various soft-thresholding powers. (B) The mean connectivity for various soft-thresholding powers. (C) Histogram of connectivity distribution (β=7). (D) Checking the scale free topology (β =7).
