# Supplementary material for: DTL Is a Prognostic Biomarker and Promotes Bladder Cancer Progression through Regulating the AKT/mTOR axis
Source: Oxid Med Cell Longev. 2022 Jan 21;2022:3369858. doi: 10.1155/2022/3369858 (PMC8799954; doi:10.1155/2022/3369858)
Supplement: Supplementary 2 — Supplementary Table 1: hub genes in the brown module. Supplementary Table 2: ssGSEA enrichment analysis in E-MTAB-4321. Supplementary Table 3: ssGSEA enrichment analysis in GSE13507. Supplementary Table 4: ssGSEA enrichment analysis in GSE32894. Supplementary Table 5: ssGSEA enrichment analysis in TCGA-BLCA. [file 3369858.f2.docx]

**Table S1** Hub genes in brown module

| Number | Hub genes |
| --- | --- |
| 1 | CDT1 |
| 2 | CCNE2 |
| 3 | STMN1 |
| 4 | HMGB2 |
| 5 | C16orf59 |
| 6 | RAD54L |
| 7 | NUF2 |
| 8 | CDCA2 |
| 9 | KIAA0101 |
| 10 | TUBA1C |
| 11 | CDC20 |
| 12 | NDC80 |
| 13 | CENPF |
| 14 | CHEK1 |
| 15 | CSE1L |
| 16 | CDKN3 |
| 17 | CENPM |
| 18 | RAD51AP1 |
| 19 | ZWINT |
| 20 | PBK |
| 21 | EXO1 |
| 22 | CHAF1B |
| 23 | C15orf42 |
| 24 | MTHFD2 |
| 25 | CIT |
| 26 | AURKA |
| 27 | MCM2 |
| 28 | CDCA5 |
| 29 | AURKB |
| 30 | KIF2C |
| 31 | TOP2A |
| 32 | C9orf48 |
| 33 | ASF1B |
| 34 | KIF20A |
| 35 | TROAP |
| 36 | C9orf140 |
| 37 | DKFZp762E1312 |
| 38 | MCM10 |
| 39 | WDR51A |
| 40 | EZH2 |
| 41 | CDCA8 |
| 42 | BLM |
| 43 | UBE2T |
| 44 | MPHOSPH1 |
| 45 | CCNB1 |
| 46 | CENPE |
| 47 | FOXM1 |
| 48 | KIF23 |
| 49 | CENPN |
| 50 | RFC4 |
| 51 | CDC25C |
| 52 | NUSAP1 |
| 53 | C1orf112 |
| 54 | PRC1 |
| 55 | FAM64A |
| 56 | SGOL1 |
| 57 | MELK |
| 58 | CDC25A |
| 59 | BUB1B |
| 60 | MKI67 |
| 61 | NUDT1 |
| 62 | BUB1 |
| 63 | C13orf3 |
| 64 | CDCA3 |
| 65 | ANLN |
| 66 | POLQ |
| 67 | CDC7 |
| 68 | ESPL1 |
| 69 | CENPL |
| 70 | UBE2C |
| 71 | DTL |
| 72 | CEP55 |
| 73 | CDC2 |
| 74 | DLG7 |
| 75 | NCAPG |
| 76 | CKAP2L |
| 77 | PTTG1 |
| 78 | C17orf53 |
| 79 | FEN1 |
| 80 | OIP5 |
| 81 | ORC1L |
| 82 | PKMYT1 |
| 83 | SPAG5 |
| 84 | UCK2 |
| 85 | C18orf24 |
| 86 | DCC1 |
| 87 | E2F2 |
| 88 | HMMR |
| 89 | PRR11 |
| 90 | UHRF1 |
| 91 | CCNA2 |
| 92 | C1orf135 |
| 93 | TTK |
| 94 | PLK4 |
| 95 | FLJ20105 |
| 96 | TPX2 |
| 97 | C1orf175 |
| 98 | KIF11 |
| 99 | TRIP13 |
| 100 | KIF4A |
| 101 | TUBA1B |
| 102 | E2F1 |
| 103 | CENPA |
| 104 | CCNB2 |
| 105 | TK1 |
| 106 | STIL |
| 107 | KIF14 |
| 108 | CDC45L |
| 109 | KIF15 |
| 110 | GINS2 |
| 111 | RNASEH2A |
| 112 | IQGAP3 |
| 113 | SPC25 |
| 114 | ASPM |

|  | logFC | AveExpr | t | P.Value | adj.P.Val | B |
| --- | --- | --- | --- | --- | --- | --- |
| COAGULATION | -0.03236 | -0.01418 | -5.90897 | 6.55E-09 | 4.94E-08 | 8.81758 |
| REACTIVE_OXYGEN_SPECIES_PATHWAY | -0.01512 | 0.106995 | -5.84528 | 9.38E-09 | 5.86E-08 | 8.468124 |
| INTERFERON_ALPHA_RESPONSE | -0.04039 | 0.326728 | -5.73556 | 1.73E-08 | 9.59E-08 | 7.873901 |
| TGF_BETA_SIGNALING | -0.02018 | 0.260539 | -5.62706 | 3.13E-08 | 1.56E-07 | 7.295869 |
| APOPTOSIS | -0.0161 | 0.173086 | -4.96156 | 9.74E-07 | 4.39E-06 | 3.96506 |
| P53_PATHWAY | -0.01301 | 0.246692 | -4.94531 | 1.05E-06 | 4.39E-06 | 3.888426 |
| HEDGEHOG_SIGNALING | -0.03426 | -0.07309 | -4.46984 | 9.79E-06 | 3.50E-05 | 1.747359 |
| PANCREAS_BETA_CELLS | -0.0251 | 0.248407 | -4.21536 | 2.98E-05 | 9.32E-05 | 0.683135 |
| HEME_METABOLISM | -0.00539 | 0.143679 | -3.58838 | 0.000367 | 0.00108 | -1.68978 |
| FATTY_ACID_METABOLISM | -0.0101 | 0.13831 | -3.53463 | 0.000448 | 0.001245 | -1.87644 |
| MYOGENESIS | -0.01509 | -0.00125 | -3.44947 | 0.000611 | 0.001609 | -2.16674 |
| BILE_ACID_METABOLISM | -0.01468 | -0.16122 | -3.36742 | 0.00082 | 0.001952 | -2.44005 |
| ALLOGRAFT_REJECTION | -0.01502 | 0.054242 | -3.33869 | 0.000908 | 0.002034 | -2.53426 |
| ADIPOGENESIS | -0.00693 | 0.261761 | -3.33001 | 0.000936 | 0.002034 | -2.5626 |
| PROTEIN_SECRETION | -0.00764 | 0.371455 | -3.31745 | 0.000978 | 0.002037 | -2.60343 |
| TNFA_SIGNALING_VIA_NFKB | -0.01312 | 0.07784 | -3.17853 | 0.001576 | 0.003153 | -3.04535 |
| KRAS_SIGNALING_DN | -0.01066 | -0.15641 | -2.75125 | 0.006162 | 0.010625 | -4.29054 |
| APICAL_JUNCTION | -0.00622 | 0.118984 | -2.30057 | 0.021846 | 0.035235 | -5.41499 |
| APICAL_SURFACE | 0.01324 | 0.049366 | 2.608618 | 0.009376 | 0.015626 | -4.66751 |
| PEROXISOME | 0.012403 | 0.173934 | 2.827205 | 0.004893 | 0.008737 | -4.08184 |
| MITOTIC_SPINDLE | 0.008414 | 0.208235 | 2.832006 | 0.004821 | 0.008737 | -4.06847 |
| DNA_REPAIR | 0.00763 | 0.233717 | 3.084429 | 0.002158 | 0.004149 | -3.33442 |
| MYC_TARGETS_V2 | 0.015833 | 0.098361 | 3.433689 | 0.000647 | 0.001618 | -2.21979 |
| SPERMATOGENESIS | 0.011947 | 0.064321 | 4.29455 | 2.12E-05 | 7.07E-05 | 1.008105 |
| GLYCOLYSIS | 0.015901 | 0.103257 | 4.674052 | 3.85E-06 | 1.48E-05 | 2.642762 |
| MYC_TARGETS_V1 | 0.012441 | 0.505198 | 5.899335 | 6.92E-09 | 4.94E-08 | 8.764502 |
| UNFOLDED_PROTEIN_RESPONSE | 0.011835 | 0.318034 | 6.993467 | 9.09E-12 | 9.09E-11 | 15.25639 |
| MTORC1_SIGNALING | 0.026998 | 0.243105 | 8.700462 | 5.37E-17 | 6.72E-16 | 27.11945 |
| UV_RESPONSE_UP | 0.022191 | 0.179637 | 10.73661 | 3.10E-24 | 5.16E-23 | 43.63242 |
| G2M_CHECKPOINT | 0.041351 | 0.22801 | 14.56026 | 5.23E-40 | 1.31E-38 | 79.75082 |
| E2F_TARGETS | 0.098253 | 0.092424 | 20.10362 | 1.38E-65 | 6.89E-64 | 138.4783 |

**Table S2** ssGSEA enrichment analysis in E-MTAB-4321

**Table S3** ssGSEA enrichment analysis in GSE13507

|  | logFC | AveExpr | t | P.Value | adj.P.Val | B |
| --- | --- | --- | --- | --- | --- | --- |
| UV_RESPONSE_DN | -0.01936 | 0.087528 | -5.35669 | 2.77E-07 | 3.46E-06 | 5.52798 |
| ADIPOGENESIS | -0.02049 | 0.282583 | -4.81414 | 3.29E-06 | 2.06E-05 | 3.129925 |
| HYPOXIA | -0.01656 | 0.08765 | -4.7305 | 4.74E-06 | 2.63E-05 | 2.777254 |
| KRAS_SIGNALING_UP | -0.02628 | 0.076527 | -4.38594 | 2.04E-05 | 9.26E-05 | 1.374907 |
| HEME_METABOLISM | -0.00858 | 0.152096 | -4.33745 | 2.49E-05 | 0.000104 | 1.184261 |
| APOPTOSIS | -0.01853 | 0.267011 | -4.25543 | 3.47E-05 | 0.000133 | 0.865633 |
| IL6_JAK_STAT3_SIGNALING | -0.02592 | -0.03733 | -4.09356 | 6.60E-05 | 0.000236 | 0.251276 |
| MYOGENESIS | -0.03379 | -0.00241 | -4.04604 | 7.95E-05 | 0.000261 | 0.074591 |
| COAGULATION | -0.0173 | 0.01406 | -4.03319 | 8.35E-05 | 0.000261 | 0.027104 |
| WNT_BETA_CATENIN_SIGNALING | -0.01795 | 0.107414 | -3.82261 | 0.000186 | 0.000547 | -0.73318 |
| TGF_BETA_SIGNALING | -0.01782 | 0.231806 | -3.68133 | 0.000313 | 0.000869 | -1.22404 |
| APICAL_JUNCTION | -0.01374 | 0.130048 | -3.28045 | 0.001261 | 0.00332 | -2.5302 |
| ANGIOGENESIS | -0.0275 | -0.11073 | -3.02465 | 0.002882 | 0.006863 | -3.29442 |
| BILE_ACID_METABOLISM | -0.01351 | -0.08005 | -2.68996 | 0.007871 | 0.017889 | -4.20983 |
| PI3K_AKT_MTOR_SIGNALING | -0.00562 | 0.190406 | -2.43868 | 0.015787 | 0.03432 | -4.83232 |
| APICAL_SURFACE | -0.01046 | -0.04166 | -2.27908 | 0.023927 | 0.046014 | -5.19817 |
| PEROXISOME | 0.009584 | 0.211556 | 2.301792 | 0.022581 | 0.045161 | -5.14752 |
| DNA_REPAIR | 0.010661 | 0.379475 | 2.394429 | 0.017753 | 0.036985 | -4.93607 |
| GLYCOLYSIS | 0.013794 | 0.161201 | 3.044733 | 0.002706 | 0.006766 | -3.23642 |
| MYC_TARGETS_V1 | 0.024082 | 0.539141 | 4.638399 | 7.05E-06 | 3.53E-05 | 2.394345 |
| MTORC1_SIGNALING | 0.021525 | 0.362794 | 4.817058 | 3.25E-06 | 2.06E-05 | 3.142309 |
| UV_RESPONSE_UP | 0.014041 | 0.197216 | 4.851128 | 2.79E-06 | 2.06E-05 | 3.287378 |
| UNFOLDED_PROTEIN_RESPONSE | 0.022878 | 0.397948 | 5.220016 | 5.25E-07 | 5.25E-06 | 4.906396 |
| MYC_TARGETS_V2 | 0.038443 | 0.330414 | 5.936663 | 1.63E-08 | 2.72E-07 | 8.288035 |
| E2F_TARGETS | 0.121963 | 0.189995 | 14.55936 | 1.54E-31 | 3.85E-30 | 60.90222 |
| G2M_CHECKPOINT | 0.073767 | 0.240164 | 16.11413 | 7.40E-36 | 3.70E-34 | 70.82196 |

**Table S4** ssGSEA enrichment analysis in GSE32894

|  | logFC | AveExpr | t | P.Value | adj.P.Val | B |
| --- | --- | --- | --- | --- | --- | --- |
| P53_PATHWAY | -0.05873 | 0.123235 | -6.66937 | 1.19E-10 | 1.48E-09 | 13.04546 |
| TGF_BETA_SIGNALING | -0.06962 | 0.110342 | -5.73256 | 2.36E-08 | 2.36E-07 | 7.876529 |
| ESTROGEN_RESPONSE_EARLY | -0.03396 | 0.105377 | -4.59681 | 6.26E-06 | 4.47E-05 | 2.475185 |
| ADIPOGENESIS | -0.02794 | 0.101047 | -4.06234 | 6.17E-05 | 0.000385 | 0.29121 |
| APOPTOSIS | -0.02377 | 0.086503 | -2.59908 | 0.009796 | 0.030614 | -4.4123 |
| MYC_TARGETS_V1 | 0.0362 | 0.110055 | 2.566147 | 0.010755 | 0.031633 | -4.49568 |
| EPITHELIAL_MESENCHYMAL_TRANSITION | 0.06378 | 0.024124 | 2.631676 | 0.008924 | 0.029745 | -4.32879 |
| GLYCOLYSIS | 0.029684 | 0.106923 | 3.259578 | 0.00124 | 0.00443 | -2.52784 |
| COMPLEMENT | 0.043577 | 0.07152 | 3.310326 | 0.001042 | 0.004009 | -2.36655 |
| INFLAMMATORY_RESPONSE | 0.070474 | 0.013902 | 3.497452 | 0.000539 | 0.002245 | -1.75181 |
| SPERMATOGENESIS | 0.021462 | 0.056492 | 3.543535 | 0.000456 | 0.002072 | -1.59563 |
| HEDGEHOG_SIGNALING | 0.063704 | 0.024729 | 3.582946 | 0.000395 | 0.001973 | -1.46056 |
| UV_RESPONSE_UP | 0.025062 | 0.081424 | 3.754172 | 0.000208 | 0.001154 | -0.85785 |
| MITOTIC_SPINDLE | 0.038165 | 0.086296 | 5.086966 | 6.33E-07 | 5.27E-06 | 4.683406 |
| MTORC1_SIGNALING | 0.083144 | 0.10149 | 7.040174 | 1.25E-11 | 2.08E-10 | 15.25375 |
| G2M_CHECKPOINT | 0.12419 | 0.094392 | 11.50019 | 1.03E-25 | 2.59E-24 | 47.33405 |
| E2F_TARGETS | 0.282759 | 0.098552 | 15.96663 | 2.99E-42 | 1.50E-40 | 85.22692 |

**Table S5** ssGSEA enrichment analysis in TCGA-BLCA

|  | logFC | AveExpr | t | P.Value | adj.P.Val | B |
| --- | --- | --- | --- | --- | --- | --- |
| XENOBIOTIC_METABOLISM | -0.0253 | 0.131393 | -8.22514 | 2.53E-15 | 2.53E-14 | 23.27565 |
| ADIPOGENESIS | -0.01429 | 0.379722 | -7.64019 | 1.51E-13 | 1.08E-12 | 19.24118 |
| ESTROGEN_RESPONSE_EARLY | -0.01997 | 0.205812 | -6.81451 | 3.34E-11 | 1.86E-10 | 13.93477 |
| FATTY_ACID_METABOLISM | -0.01743 | 0.196895 | -6.44932 | 3.13E-10 | 1.56E-09 | 11.74192 |
| MYOGENESIS | -0.02869 | 0.030815 | -6.15297 | 1.79E-09 | 8.14E-09 | 10.03515 |
| ANDROGEN_RESPONSE | -0.01625 | 0.330907 | -5.69461 | 2.34E-08 | 9.01E-08 | 7.528193 |
| NOTCH_SIGNALING | -0.02094 | 0.312619 | -5.44208 | 9.02E-08 | 3.01E-07 | 6.217923 |
| BILE_ACID_METABOLISM | -0.01282 | -0.0898 | -4.68568 | 3.79E-06 | 1.16E-05 | 2.606188 |
| IL6_JAK_STAT3_SIGNALING | -0.02791 | 0.086897 | -4.67618 | 3.96E-06 | 1.16E-05 | 2.563916 |
| CHOLESTEROL_HOMEOSTASIS | -0.01559 | 0.308649 | -4.61168 | 5.33E-06 | 1.48E-05 | 2.278732 |
| P53_PATHWAY | -0.0138 | 0.383685 | -4.33087 | 1.86E-05 | 4.66E-05 | 1.079141 |
| PEROXISOME | -0.01398 | 0.20625 | -3.99246 | 7.73E-05 | 0.000184 | -0.27473 |
| COAGULATION | -0.0112 | 0.024448 | -2.86306 | 0.004408 | 0.009184 | -4.04241 |
| ESTROGEN_RESPONSE_LATE | -0.01287 | 0.230176 | -2.79989 | 0.005351 | 0.010701 | -4.21816 |
| ANGIOGENESIS | -0.02041 | 0.012637 | -2.50556 | 0.012608 | 0.023348 | -4.98703 |
| INTERFERON_GAMMA_RESPONSE | 0.016222 | 0.209218 | 2.224366 | 0.026661 | 0.044435 | -5.64416 |
| INFLAMMATORY_RESPONSE | 0.016985 | -0.0806 | 2.240794 | 0.025567 | 0.044082 | -5.60786 |
| PROTEIN_SECRETION | 0.006317 | 0.463325 | 2.39697 | 0.016973 | 0.030308 | -5.24981 |
| INTERFERON_ALPHA_RESPONSE | 0.019362 | 0.371841 | 2.744241 | 0.006328 | 0.012169 | -4.36984 |
| COMPLEMENT | 0.015275 | 0.150807 | 3.47557 | 0.000563 | 0.001225 | -2.1446 |
| PANCREAS_BETA_CELLS | 0.019219 | -0.2403 | 3.582537 | 0.000381 | 0.000865 | -1.77756 |
| UNFOLDED_PROTEIN_RESPONSE | 0.007524 | 0.470738 | 4.500745 | 8.81E-06 | 2.32E-05 | 1.796656 |
| MYC_TARGETS_V2 | 0.027992 | 0.367993 | 5.656647 | 2.88E-08 | 1.03E-07 | 7.32797 |
| UV_RESPONSE_UP | 0.010163 | 0.314602 | 5.947388 | 5.79E-09 | 2.41E-08 | 8.890529 |
| MITOTIC_SPINDLE | 0.013839 | 0.425965 | 7.122112 | 4.72E-12 | 2.95E-11 | 15.85645 |
| MYC_TARGETS_V1 | 0.016616 | 0.612434 | 7.886519 | 2.77E-14 | 2.31E-13 | 20.9134 |
| SPERMATOGENESIS | 0.018893 | -0.06848 | 8.822911 | 3.13E-17 | 3.91E-16 | 27.61743 |
| MTORC1_SIGNALING | 0.023621 | 0.431681 | 9.02566 | 6.73E-18 | 1.12E-16 | 29.13762 |
| G2M_CHECKPOINT | 0.047573 | 0.379328 | 15.08525 | 2.60E-41 | 6.50E-40 | 82.73433 |
| E2F_TARGETS | 0.081907 | 0.330059 | 17.47204 | 1.22E-51 | 6.11E-50 | 106.4494 |
